# Cost-Effectiveness Analysis of a Mobile-Based Intervention for Patients with Type 2 Diabetes Mellitus

**DOI:** 10.1155/2021/8827629

**Published:** 2021-07-01

**Authors:** Jing Li, Li Sun, Yabing Hou, Liming Chen

**Affiliations:** ^1^National Health Commission Key Laboratory of Hormones and Development, Tianjin Key Laboratory of Metabolic Diseases, Chu Hsien-I Memorial Hospital & Tianjin Institute of Endocrinology, Tianjin Medical University, No 6, Huanruibei Road, Beichen District, Tianjin 300134, China; ^2^School of Nursing, Tianjin Medical University, Tianjin 300070, China; ^3^School of Public Health, Tianjin Medical University, No 22, Qixiangtai Road, Heping District, Tianjin 300070, China

## Abstract

**Objective:**

The aim of this study was to evaluate the cost effectiveness of a mobile-based intervention for patients with type 2 diabetes mellitus (T2DM) and compare it with the usual management mode.

**Method:**

A total of 215 patients with T2DM in a tertiary-care hospital specific to diabetes were selected as the study population. This study was conducted from January 1, 2019 to January 1, 2020. Of the 215 patients, 130 were randomly assigned to the mHealth group and 85 were assigned to the usual care group. IBM SPSS 25.0 software was used for descriptive statistics, *t* tests, chi-square tests, and correlation analyses. Haemoglobin A1c (HbA1c) was the effectiveness parameter adopted. Cost-effectiveness analyses were performed, and incremental cost-effectiveness ratios (ICERs) were calculated.

**Results:**

Of the 215 patients with T2DM, the proportion of male patients was 66.0%. The mean age of the patients was 47.2 (SD 9.95). Differences in baseline information were not statistically significant between the two groups (*P* > 0.05). At the 3-, 6-, and 12-month follow-ups, the mHealth group reported higher control rates of HbA1c than the usual care group, 67.9% versus 46.2% (*P* < 0.001), 72.4% versus 45.4% (*P* < 0.001), and 74.6% versus 47.1% (*P* < 0.001), respectively. The value of HbA1c was positively related to total patient cost, material fee, Western medicine fee, and hospitalization expenses (*P* < 0.05), with correlation coefficients of 0.202, 0.200, 0.172, and 0.183, respectively. The costs of the mHealth group and usual care group were CNY¥ 1169.76 and CNY¥ 1775.44 per patient/year, respectively. The incremental cost of the mHealth intervention was CNY¥ −605.68 per patient/year. The ICER was CNY¥ −22.02 per patient/year.

**Conclusion:**

Compared with the usual care mode, the mHealth management model for patients with T2DM improved the control rate of HbA1c, and the mHealth management mode had better cost effectiveness.

## 1. Introduction

With the development of the social economy, the number of patients with diabetes mellitus (DM) in the world is increasing. According to estimates by the World Health Organization, as of November 2016, the number of adults with DM worldwide had increased to 422 million [1]. During the past 30 years, the prevalence of DM in China has been severe. According to an epidemiological survey conducted by the National Health and Family Planning Commission, there are approximately 3,000 new cases of DM in China every day, an increase of approximately 200,000 patients every year [2]. The number of people with DM in China will reach 42.3 million by 2030 and is expected to reach 143 million in 2035, ranking first in the world [1].

A large population base, high prevalence, and high incidence of complications impose a heavy economic burden on patients, their families, and society. It is estimated that 2.5% to 15% of the annual health budget is devoted to the treatment of DM and its complications. Globally, 2.49 billion disability-adjusted life years were lost in 2010, of which 47 million (or 1.9%) were lost due to DM [3]. The Total Economic Burden of Chronic Noncommunicable Diseases in China study showed that the total economic burden of DM in 2003 reached 23.706 billion, accounting for 1.97% of all noncommunicable disease burdens [4]. In 2004, the direct medical cost of DM accounted for 7.2% to 10.1% of the total medical and health expenses in the United States and Europe, which was close to or even exceeded some developed countries [5]. The cost of DM treatment increased to 200 billion in 2007 [6]. In China, the total medical cost of treating type 2 diabetes and its complications is 20.860 billion per year in urban areas, accounting for 4.38% of the total medical and health expenses and 35% to 73% of the annual per capita income of urban residents. Among them, the cost of treating complications was 16.451 billion, which has become a major source of economic burden [6]. In 2010, the Chinese Diabetes Association showed that the DM direct health expenditure in China was 173.4 billion, accounting for 13% of the national health expenditure [4]. The incidence of DM is increasing rapidly, and the prevention and control of DM is poor, which is bound to cause a growing economic burden of diabetes and uneven demand for health resources and diabetes health services. National health planning and resource allocation will face serious challenges. To explore prevention and treatment measures to reduce the incidence of diabetes, decreasing the incidence of complications and medical costs has become the focus of global health and medical management.

For patients with DM, many interventions can improve their health outcomes, regardless of what type of diabetes they may have, including glycaemic control combined with diet, physical activity, and medication. However, most studies have focused on pharmacoeconomic evaluations of drug treatment. Although the costs and effects of different medication regimens to screen cost-effective medication regimens have been measured, there are few reports on the health economics evaluation of intervention management models, and the evaluation of medical consequences in research involves mostly clinical biochemical indicators. Therefore, it is necessary to conduct health economic evaluations of diabetes interventions.

With the development of Internet of Things technology, health management modes based on network platforms or mobile intelligent tools have received increased attention. In a management model mediated by mobile intelligence, the main purpose of diabetes management is to strengthen self-management, which makes up for the deficiency of the traditional model. Human and material resources were effectively utilized to help medical staff better monitor the changes in patients' condition and improve compliance. Patients also took the initiative to participate in their own health management. Their self-efficacy was improved, and social support was increased. The aim of this study was to evaluate the cost effectiveness of a mobile-based intervention for patients with type 2 diabetes mellitus (T2DM) and compare it with the usual management mode.

## 2. Methods

### 2.1. Subjects

This study was conducted in a tertiary-care hospital specific to diabetes in Tianjin, China, from January 1, 2019, to January 1, 2020. The study included patients of both sexes aged 18 years and above with T2DM registered in the clinical database for enrolment and follow-up of patients with DM living in the territories served by Community Health Care. All patients who registered in the clinical database between November 1, 2018, and December 31, 2018, were included in our source population. Then, we excluded patients who had no interest in participating in this study and who had no smartphone. Finally, we identified 215 patients with T2DM and randomly divided them into an mHealth group (130) and a usual care group (85) by drawing lots. After obtaining informed consent, the patients in the mHealth group downloaded the app under the guidance of doctors or nurses.

### 2.2. Interventions

We followed the intervention methods of Li et al. [7]. Patients in the usual care group received standard medical care every three months. Patients in the mHealth group received a mobile-based intervention. This was a continuous medical service model running through and outside the hospital. It used mobile Internet, Internet of Things, and cloud computing to establish a health-care team of doctors, nurses, health educators, and dietitians. This model extended medical services from hospitals to families. The diabetes data management system and app were designed to provide hardware and software support for this intervention. The service objects of the diabetes data management system were doctors, nurses, and health-care providers in different medical institutions. Its functional modules included outpatient management, patient management, appointment management, out-of-hospital follow-up, patient reminders, and online consultation. Information can be shared between different medical institutions. The functions of the app include authentication, appointment registration, electronic medical record access, medication reminders, results checks, blood glucose monitoring and threshold alarms, diet and exercise monitoring and suggestions, health education, telephone follow-up, real-time communication with the management team, and peer support and communication. In this medical service mode, doctors can analyse and process real-time monitoring data and strengthen the blood glucose management of patients. Patients used the app to communicate with medical staff in real time. Patients can also upload the self-test values of blood glucose, diet, exercise, and other out-of-hospital data through the app. Meanwhile, the in-hospital visit data of patients can also be transmitted to the app. Medical staff can check patient data at any time, fully and accurately understand the patient's condition, and provide a basis for diagnosis and treatment decisions. Patients and the health-care team communicated in real time, which was an effective way to determine patients' problems relating to blood glucose monitoring, medication, diet, or exercise. Then, the health-care team proposed solutions to these problems. This model can also reduce the number of hospital visits and save time and economic costs for patients.

### 2.3. Outcome Definition

The health outcome was the control rate of HbA1c at baseline and at the 3-, 6-, and 12-month follow-ups. We identified the control rate according to guidelines for the prevention and control of type 2 diabetes in China (2017 edition) [8]. The control objective was defined as HbA1c < 7%. To calculate the costs involved in the care of these patients, total patient cost, registration fee, material fee, treatment fee, Western medicine fee, laboratory fee, Chinese patent drug fee, and hospitalization expenses were considered.

### 2.4. Statistical Analysis

We present categorical variables as numbers (percentages) and continuous variables as means (standard deviations, SD). Descriptive statistics were used to analyse patient demographics. Binary or categorical outcome measures were analysed using the chi-square test, and continuous measures were analysed using the *t*-test or a nonparametric equivalent (e.g., Wilcoxon rank test). We used correlation analysis to explore the associations between HbA1c and cost. We determined statistical significance using a two-tailed *P* value of <0.05. All of the statistical analyses were carried out using IBM SPSS version 25.0.

Cost-effectiveness analysis (CEA) is a method of comparing decision alternatives in which both the costs and the effects are taken into account in a systematic way [9]. For the calculation, the mean of the total annual cost of each group in the follow-up period compared with the difference in HbA1c between follow-up and baseline was considered. The incremental cost-effectiveness ratio (ICER) was calculated as ICER = △*C*/△*E*.

## 3. Results

### 3.1. Patient Demographics

Of the 215 patients with T2DM, 130 were randomly assigned to the mHealth group and 85 were assigned to the usual care group. The study population consisted mainly of males, with a proportion of 66.0% (142/215). The mean age of the patients was 47.2 (SD 9.95) years. In the mHealth group, the proportion of male patients was 67.7% (88/130), with a mean age of 47.5 (SD 9.88) years. In the usual care group, the proportion of male patients was 63.5% (54/85), with a mean age of 46.7 (SD 10.11) years. Differences in baseline information were not statistically significant between the two groups (*P* > 0.05).

### 3.2. Control Rates of HbA1c

At baseline, the control rates of HbA1c in the mHealth and usual care groups were 43.7% and 44.1% (*P*=0.61), respectively. At the 3-, 6-, and 12-month follow-ups, the mHealth group reported higher control rates of HbA1c than the usual care group, which were 67.9% versus 46.2% (*P* < 0.001), 72.4% versus 45.4% (*P* < 0.001), and 74.6% versus 47.1% (*P* < 0.001), respectively. Differences in the control rates between the two groups at these three follow-up sessions were 21.7%, 27.0%, and 27.5%, respectively.

### 3.3. Cost-Effectiveness Analysis

Table 1 presents the results of the cost comparison between the mHealth group and usual care group at the 12-month follow-up. Between the two groups, there were significant differences in material fees, treatment fees, Western medicine fees, laboratory fees, Chinese patent drug fees, hospitalization expenses, and total patient costs (*P* < 0.01). Among them, the costs of treatment and laboratory work in the mHealth group were higher than those in the usual care group (*P* < 0.001), whereas the other costs were lower than those in the usual care group (*P* < 0.01). In each group, there were two subgroups, including HbA1c control or HbA1c control. The costs of both subgroups were not significantly different (*P* > 0.05).

**Figure 1:**
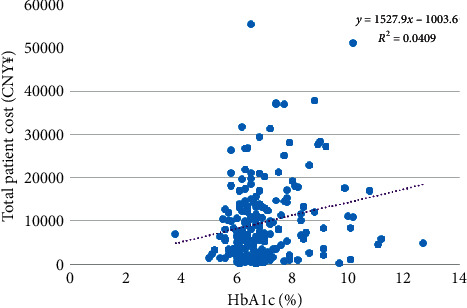
The linear associations between HbA1c and total patient cost.

Table 2 indicates the correlation between HbA1c and cost. The value of HbA1c was positively related to total patient cost, material fee, Western medicine fee, and hospitalization expenses (*P* < 0.05), with correlation coefficients of 0.202, 0.200, 0.172, and 0.183, respectively. There were significant negative correlations among treatment fee, laboratory fee, and HbA1c (*P* < 0.05), with correlation coefficients of −0.289 and −0.148. The linear association between HbA1c and total patient cost is shown in Figure 1. The fitting curve equation of correlation was *y* = 1527.9*x*  − 1003.6, with adjusted *R*^*2*^ = 4.09%.

Table 3 shows the cost-effectiveness analysis comparison of the mHealth group and usual care group. In the mHealth group, the cost was CNY¥ 1169.76 per patient/year, whereas in the usual care group, the cost was CNY¥ 1775.44 per patient/year. The incremental cost of mHealth intervention was CNY¥ −605.68 per patient/year. In the mHealth group, the cost of unit HbA1c control rate was CNY¥ 1541.19 per patient/year, whereas in the usual care group, the cost of unit HbA1c control rate was CNY¥ 3769.50 per patient/year. These values showed an ICER of CNY¥ −22.02 per patient/year; that is, the mHealth intervention was able to save CNY¥ 22.02 per patient/year for the health service.

## 4. Discussion

In this study, cost differences between the mHealth group and usual care group at the 12-month follow-up were observed. We found that the costs of material fee, Western medicine fee, Chinese patent drug fee, hospitalization expenses, and total patient costs in the mHealth group were significantly lower than those in the usual care group. The incremental cost of the mHealth intervention was CNY¥ −605.68 per patient/year. The ICER was CNY¥ −22.02 per patient/year; that is, the mHealth intervention was able to save CNY¥ 22.02 per patient/year for the health service.

The purpose of diabetes self-management is to enable diabetic patients to acquire knowledge and skills in disease control, to prevent complications and have better glycaemic control, and to change negative attitudes towards disease treatment to improve quality of life within the economically feasible range. The cost-effect analysis of intensive treatment for diabetic patients was based on the assumption that intensive treatment might be more expensive than nonintensive treatment, but intensive treatment reduced the cost of chronic complications [10]. The entire cost-effectiveness analysis suggested that intensive treatment was better. The modern medical model incorporated the quality of life assessment into the evaluation of the efficacy for diabetic patients. Quality of life was also closely related to clinical economic analysis. Sidorov et al. [11] studied the impact of disease management plans on patients' medical costs and showed that the average compensation for patients who did not participate in self-management was US $502.48/person/month and that of patients who joined self-management was US $394.62/person/month; the average saved total medical compensation was US $1294.32/person/year. A community diabetes prevention project carried out by Johansson et al. [12] in Sweden also achieved a good cost-effectiveness ratio. According to the test results, in actual implementation, the cost of the project to obtain each QALY was £2.09, far less than the reference value of £2,000. Wier [13] and Sagarara [14] used randomized controlled trials to prove that intervention projects for patients with type 2 diabetes have good economic benefits. Gillespie P et al. [15] evaluated 437 type 1 diabetic patients with a classification structure education management model for one and a half years. The results showed that the average total cost of the earlier routine management group for diabetic patients under the grouping model was reduced by £772. Although foreign studies have analysed various aspects of cost benefits and cost effectiveness, the general social conditions and long-term effects of the subsequent project are rarely reported, and the results of the overall medical cost benefit are not accurate [16].

Increasing demand for health care and rapid growth of health-care costs have become the main problems hindering the development of medical and health care. To alleviate the contradiction between limited resources and unlimited demand, economic evaluation of different interventions has become particularly important. When the pros and cons of the prevention plan are similar and the two plans are similar in cost, the plan with a better effect is selected. When the two plans have similar effects, the plan with the lower cost investment is selected. Only the cost is considered, and the effect is not considered. Considering the effect, regardless of the cost, the intervention plan will cause a waste of medical resources. For this reason, domestic and foreign scholars have launched corresponding studies [17, 18], but most of these studies are pharmacoeconomic evaluations of the drug treatment of type 2 diabetes. Although the costs and effects of different medication regimens to screen cost-effective medication regimens have been measured, there are few reports on the health economics evaluation of intervention management models, and the evaluation of medical consequences in research involves mostly clinical biochemical indicators. There are few economic evaluations combined with improving the quality of life of patients and families.

Many researchers have conducted cost-effectiveness analyses of diabetes intervention projects. The Diabetes Initiative was a comprehensive diabetes self-management project implemented in primary-care institutions and community institutions, covering 14 projects in urban, rural, and border areas throughout the United States. The main forms of intervention were regular family visits and telephone follow-ups, health consultations, health education, and the establishment of communication groups or clubs to help patients self-manage diabetes. Bronson et al. [19] conducted health economics on the plans of four areas of the project. The results showed that the incremental cost-effectiveness rate of the project was far below the reference threshold. A community diabetes education and self-management project carried out in the UK in 2004 [20] also achieved a very good cost-effectiveness ratio. The results of the intervention study by Heinrich et al. [21] showed that compared with the conventional management group, the intervention group only needed US $1,080 to obtain unit life years. However, many studies mostly used extended life years as an indicator of effect evaluation. Due to the short intervention time in this study, the occurrence, development, and death of patients' complications cannot be grasped specifically, so the intervention effect of the mHealth model was judged based on the control rate of HbA1c.

In this study, the mobile-based intervention was a continuous medical service model running through and outside the hospital. This model extends medical services from hospitals to families. The health-care team, including doctors, nurses, health educators, and dietitians, provides continuous medical service for patients with T2DM. Based on the diabetes data management system, patient information can be shared between different medical institutions, which can reduce the cost of duplicate examinations. Patients and the health-care team can communicate in real time on the app to solve the problems of blood glucose monitoring, medication, diet, or exercise, which can also reduce the number of hospital visits and hospitalizations and save time and economic costs for patients. In this study, we used the intervention effect evaluation index to conduct an economic evaluation of the mobile-based management model. Through the analysis and comparison of the cost and management effect of different intervention programmes, the best intervention strategies are screened out to provide a clinical reference for choosing economical and effective intervention programmes, to promote the prevention and treatment of diabetes, to reduce the economic burden of diabetes, to make relevant medical management decisions, and to provide a basis for optimizing the allocation of health resources.

This study preliminarily verified that mobile-based intervention was more economical and effective than the usual care model based on real-world data. We chose the control rate of HbA1c as an effect indicator and calculated the ICER. This study also had several limitations. First, the intervention time was short (12 months). Therefore, we cannot evaluate the increased costs of patient complications and the change in extended life years. Second, some potential costs were not considered, such as transportation cost, work delay cost, and the workload cost of medical staff. We need to explore more quantitative calculation methods to evaluate these costs in future research.

## 5. Conclusions

This study revealed that the mobile-based intervention could improve the control rate of HbA1c, and this mHealth management mode has better cost effectiveness than the usual care mode.

## Figures and Tables

**Table 1:** Cost comparison between the mHealth group and usual care group at 12-month follow-up (CNY¥).

	*n*	Registration fee	Material fee	Treatment fee	Western medicine fee	Laboratory fee	Chinese patent drug fee	Hospitalization expenses	Total patient Cost
		Mean (SD)	Mean (SD)	Mean (SD)	Mean (SD)	Mean (SD)	Mean (SD)	Mean (SD)	Mean (SD)
Usual care group	85	355.04	302.01	18.88	7491.68	507.51	716.65	3036.29	12428.06
		(322.30)	(325.71)	(18.91)	(6922.83)	(501.28)	(719.88)	(5784.70)	(11122.32)
mHealth group	130	379.87	128.61	157.55	5099.62	811.79	225.24	951.35	7754.03
		(282.30)	(203.79)	(103.28)	(4832.08)	(395.51)	(634.72)	(2955.21)	(7118.53)
T		−0.596	4.380	−14.931	2.774	−4.718	5.124	3.071	3.441
*P*		0.552	<0.001	<0.001	0.006	<0.001	<0.001	0.003	0.001

Usual care-HbA1c uncontrol	45	377.11	348.83	18.89	7945.19	532.71	696.72	3045.30	12964.75
		(340.78)	(360.50)	(18.28)	(7255.01)	(539.55)	(634.65)	(6200.01)	(11620.74)
Usual care-HbA1c control	40	340.78	249.34	18.87	6981.48	479.15	739.08	3026.16	11824.27
		(302.54)	(276.59)	(19.82)	(6583.05)	(459.59)	(812.89)	(5357.71)	(10648.13)
t		0.668	1.414	0.005	0.638	0.489	−0.269	0.015	0.470
*P*		0.506	0.161	0.996	0.525	0.626	0.788	0.988	0.640

mHealth-HbA1c uncontrol	33	429.52	173.77	129.32	6347.86	747.45	278.60	2098.85	10205.37
		(351.12)	(225.54)	(112.33)	(6369.31)	(402.88)	(600.78)	(4472.55)	(9488.17)
mHealth-HbA1c control	97	362.97	113.25	167.15	4674.96	833.68	207.09	560.96	6920.07
		(254.67)	(194.70)	(98.79)	(4139.41)	(392.67)	(647.86)	(2112.06)	(5942.70)
t		1.171	1.480	−1.834	1.411	−1.069	0.558	1.904	1.868
*P*		0.244	0.141	0.069	0.166	0.290	0.578	0.065	0.069

**Table 2:** Correlation between HbA1c and cost (*r*).

	HbA1c	Total patient cost	Registration fee	Material fee	Treatment fee	Western medicine fee	Laboratory fee	Chinese patent drug fee
HbA1c								
Total patient cost	0.202^*∗∗*^							
Registration fee	0.062	0.797^*∗∗*^						
Material fee	0.200^*∗∗*^	0.686^*∗∗*^	0.706^*∗∗*^					
Treatment fee	−0.289^*∗∗*^	0.143^*∗*^	0.387^*∗∗*^	−0.068				
Western medicine fee	0.172^*∗*^	0.887^*∗∗*^	0.909^*∗∗*^	0.773^*∗∗*^	0.207^*∗∗*^			
Laboratory fee	−0.148^*∗*^	0.370^*∗∗*^	0.570^*∗∗*^	0.346^*∗∗*^	0.505^*∗∗*^	0.432^*∗∗*^		
Chinese patent drug fee	0.101	0.587^*∗∗*^	0.530^*∗∗*^	0.575^*∗∗*^	−0.121	0.588^*∗∗*^	0.263^*∗∗*^	
Hospitalization expenses	0.183^*∗∗*^	0.673^*∗∗*^	0.189^*∗∗*^	0.165^*∗*^	−0.057	0.265^*∗∗*^	−0.022	0.185^*∗∗*^

^*∗*^ *P* < 0.05, ^*∗∗*^*P* < 0.01.

**Table 3:** Comparison of cost-effectiveness analysis between the mHealth group and usual care group.

	HbA1c control rate (%)	Cost (CNY¥)	CEA	ICER
mHealth group	74.6%	1169.76	1541.19	−22.02
Usual care group	47.1%	1775.44	3769.50	

## Data Availability

The data belong to the funders and are not available to the public in order to protect the patient privacy.
